# The Impact of Introducing Sacubitril/Valsartan and SGLT2 Inhibitors in a Cohort of Patients with Reduced-Ejection-Fraction Heart Failure: A Real-Life Observational Study

**DOI:** 10.3390/jcm15030991

**Published:** 2026-01-26

**Authors:** Andrea López-López, Margarita Regueiro-Abel, Charigan Abou Johk-Casas, José María Vieitez-Flórez, Juliana Elices-Teja, Jorge Armesto-Rivas, Gonzalo de Urbano-Seara, Alejandro Manuel López-Pena, Carmen Cristina Álvarez-Suárez, Gema Rois-González, Germán Santamarina-Pernas, Carlos González-Juanatey

**Affiliations:** 1 Department of Cardiology, Hospital Universitario Lucus Augusti, 27002 Lugo, Spain; margarita.regueiro.abel@sergas.es (M.R.-A.); charigan.abou.jokh.casas@sergas.es (C.A.J.-C.); chemavieitez@gmail.com (J.M.V.-F.); juliana.elices.teja@sergas.es (J.E.-T.); jorge.armesto.rivas@sergas.es (J.A.-R.); gema.rois.gonzalez@sergas.es (G.R.-G.); carlos.gonzalez.juanatey@sergas.es (C.G.-J.); 2 CardioHULA Research Group, Instituto de Investigación Sanitaria de Santiago de Compostela IDIS, 27002 Lugo, Spain; 3 Faculty of Veterinary Medicine, Universidad de Santiago de Compostela, 27001 Lugo, Spain

**Keywords:** heart failure, reduced ejection fraction, neurohormonal treatment, devices, mortality

## Abstract

**Background/Objectives**: Reduced-ejection-fraction heart failure (HFrEF) constitutes a challenge due to its high morbidity and mortality. The use of sacubitril/valsartan (angiotensin receptor–neprilysin inhibitors [ARNI]) and SGLT2 inhibitors (SGLT2i) represents a change in management approach with a demonstrated association with positive ventricular remodeling and a reduction in cardiovascular events. We describe the clinical and therapeutic course of patients with HFrEF in a specialized unit, comparing two consecutive periods (2011–2016 vs. 2017–2021), with emphasis on the impact of ARNI and SGLT2i upon clinical parameters and the use of devices. **Methods**: A retrospective, longitudinal observational study was carried out in 1363 outpatients with HFrEF, with at least two years of follow-up. Clinical characteristics, treatments, the evolution of left ventricular ejection fraction (LVEF), mortality, and the use of devices (implantable cardioverter–defibrillator [ICD], cardiac resynchronization therapy [CRT]) were evaluated. **Results**: A total of 1363 patients were analyzed, showing a significant therapeutic change in the 2017–2021 group with the incorporation of ARNI (40%) and SGLT2i (25%). This cohort achieved better ventricular recovery, with a significantly higher mean LVEF at one year compared to the 2011–2016 group (44.3% vs. 42.1%; *p* = 0.004). Regarding devices, ICD implantation rate decreased in the recent period (7.2% vs. 11.1%; *p* = 0.016), while CRT indication increased. Most importantly, all-cause mortality after two years fell from 9.4% to 5.9% (*p* = 0.023). Multivariate analysis confirmed that this survival improvement was independently associated with the study period (HR 1.57 for the earlier group) and was linked to the protective effect of contemporary pharmacological treatments. **Conclusions**: The systematic introduction of ARNI and SGLT2i in the treatment of HFrEF was associated with improved ventricular function, reduced need for device implantation, and lower mortality over the middle term in a real-life clinical setting.

## 1. Introduction

Reduced-ejection-fraction heart failure (HFrEF) is an important public health problem worldwide, with high morbidity and mortality and a significant impact on patient quality of life and healthcare costs [1,2,3]. Despite the advances in treatment, the prognosis remains unfavorable, particularly in the advanced stages of the disease [4]. It should be noted that heart failure is related to risk factors such as age, sex, and body mass index. With regard to BMI, obesity can cause HF to develop or worsen existing disease [5].

In the last decade, a number of drug treatments have been introduced that have substantially modified the management of HFrEF [6]. Firstly, the approval of sacubitril/valsartan, based on the results of the PARADIGM-HF trial in 2014, and subsequently the introduction of SGLT2 inhibitors (SGLT2i), as new incorporations to the pharmacological arsenal in patients with HFrEF, has led to a therapeutic paradigm shift. Their incorporation to the clinical practice guidelines starting in 2016 and 2021, respectively, has been associated with improved patient survival and a significant reduction in hospital admissions due to decompensated heart failure [7,8,9,10,11,12]. However, few longitudinal descriptive studies in the real-life setting have analyzed the impact of this change in treatment in large cohorts with a prolonged follow-up period [13]. It is essential to evaluate these changes in real-life care settings in order to understand the clinical impact of the new therapeutic strategies, beyond the controlled conditions of clinical trials.

In this context, this study aimed to describe the clinical characteristics, treatments and prognostic aspects in a large cohort of patients with HFrEF who were treated in the specialized heart failure (HF) unit between the years 2011 and 2021, comparatively evaluating two consecutive clinical periods: before (2011–2016) and after (2017–2021) the systematic introduction of sacubitril/valsartan and SGLT2i. The hypothesis was that these therapeutic changes are associated with improvement of the clinical parameters, ejection fraction, and patient survival over the middle term, as well as a decrease in the indication of devices such as implantable cardioverter–defibrillators (ICD).

### Objectives

The main objective of the present study was to describe the clinical characteristics, treatments, and prognostic aspects in a cohort of patients with HFrEF seen in a specialized HF unit between 2011 and 2021.

Specifically, the following was analyzed:

- Evolution of the therapeutic profile, especially the use of sacubitril/valsartan (angiotensin receptor–neprilysin inhibitors, ARNI) and SGLT2i in two consecutive periods (2011–2016 vs. 2017–2021).
- Evolution of left ventricular ejection fraction (LVEF) at one year of follow-up in each of the groups.
- The indication of devices such as implantable cardioverter–defibrillators (ICD) and cardiac resynchronization therapy (CRT).
- Compared mortality at two years between the two periods.

## 2. Methods

### 2.1. Study Design and Patients

A retrospective, longitudinal, single-center observational study was carried out in a cohort of consecutive patients seen in the Specialized Heart Failure Unit of the Department of Cardiology of a secondary hospital between January 2011 and December 2021. All patients had reduced-ejection-fraction heart failure (HFrEF), defined according to the current clinical guidelines of the European Society of Cardiology (ESC) [6]. A total of 1363 outpatients were included and divided into two groups according to the date of first consultation:

- Group 1: patients seen between 2011 and 2016.
- Group 2: patients seen between 2017 and 2021.

All patients had at least two years of clinical follow-up, making it possible to evaluate their evolution in terms of treatment, ventricular function and survival.

### 2.2. Data Collection

The clinical, demographic, laboratory test, electrocardiographic, echocardiographic, and pharmacological data, as well as the use of devices and mortality assessment, were recorded by the unit’s cardiologists by reviewing the patients’ electronic medical records. The ejection fraction was assessed using echocardiography (IE-33 and EPIQ-5, Philips, Bothell, WA, USA) at the start of follow-up and at the subsequent controls. All the data were entered in a database previously designed for the conduction of observational studies in the unit. Patient management was in accordance with the clinical practice guidelines available at the time of each visit.

### 2.3. Statistical Analysis

Quantitative variables were reported as the mean and standard deviation (SD), and qualitative variables were reported as frequencies and percentages. The chi-square test or Fisher exact test was used to compare qualitative variables between groups, while quantitative variables with and without a normal distribution were compared using the Student *t*-test or non-parametric tests, respectively. To calculate drug dosage over 1, only the patients who received drug treatment were taken into account.

Survival was analyzed using Kaplan–Meier curves, and comparisons between groups were made using the log-rank test. Furthermore, multivariate analyses were performed to identify independent predictors of outcomes, specifically utilizing Cox proportional-hazards regression models to estimate hazard ratios (HR) and their corresponding 95% confidence intervals (CI). The statistical analyses were performed using the IBM SPSS version 19.0 package (IBM, Armonk, NY, USA), considering a statistical significance level of *p* < 0.05.

### 2.4. Ethical Particulars

The study was carried out in abidance with the principles of the Declaration of Helsinki and Good Clinical Practice guidelines. The study protocol was approved by the Research Ethics Committee in October 2025 (approval number: 2025/378).

## 3. Results

### 3.1. Cohort Description

The study included a total of 1363 patients diagnosed with HFrEF and followed up on an outpatient basis in a specialized HF unit between the years 2011 and 2021. The patients were divided into two groups according to the date of first consultation: Group 1 (2011–2016): *n* = 759; Group 2 (2017–2021): *n* = 604.

There were no statistically significant differences between the groups in terms of mean age, sex distribution, initial LVEF, or body mass index. Creatinine levels, glomerular filtration rate, and comorbidities were similar in both groups. The patients in group 1 (2011–2016) had a higher mean systolic blood pressure (SBP), and a greater percentage of patients presented NYHA functional class III or IV, while the patients in group 2 (2017–2021) presented dyslipidemia more frequently than the other group (Table 1). All the patients were clinically stable at the time of inclusion, with no signs of acute decompensation.

### 3.2. Pharmacological Treatment at First Consultation and After One Year of Follow-Up

Patients in group 2 (2017–2021) showed a substantial change in therapeutic strategy compared to the previous years, with a significantly greater use of sacubitril/valsartan (ARNI) (42.8% vs. 0.1% in group 1) and SGLT2i (24.3% vs. 0%, respectively), in line with the current recommendations. This change was evident on the first visit and after one year (Table 2).

On comparing the proportion of angiotensin-converting enzyme inhibitors (ACEi) prescribed between the two groups at both the first visit and after one year of follow-up, a progressive transition was observed from ACEi/angiotensin receptor II antagonist (ARB) therapy towards ARNI, as indicated by the guidelines prior to 2021. This clinical pattern, where many patients started treatment with ACEi and then switched to ARNI, is coherent with the current regulatory restrictions in the first years of introduction of the drug.

The proportion of patients prescribed beta-blockers was lower in the group of patients that started consultation in 2017–2021 (although the proportion was very high in both groups), while no significant differences were observed in the indication of MRA.

### 3.3. Treatment Dose at First Consultation and After One Year of Follow-Up

To standardize the evaluation of pharmacological management, drug doses were analyzed as a proportion of the target doses established by the 2021 ESC guidelines, keeping this criteria constant regardless of the inclusion period to ensure cohort comparability. The intensity of the treatment was quantified using a ratio where the prescribed dose was divided by the maximum recommended dose; thus, a value of 1 was assigned to the achievement of the target dose, while values less than one represented sub-maximal doses and values greater than one indicated doses exceeding guideline recommendations. For the calculation of the mean and standard deviation, all values for each drug within their corresponding pharmacological group were used. In the specific case of combination therapies involving ACEi, ARB, and ARNI, individual ratios for each drug were calculated first, and then the mean of the three groups was obtained, treating them as a single pharmacological class averaged per class (Table 3).

In the group corresponding to the period 2011–2016, the initial beta-blocker doses were significantly higher than in the group corresponding to 2017–2021 (0.73 vs. 0.61; *p* < 0.001), and this difference persisted after one year of follow-up, though with an overall increase in both groups (0.89 vs. 0.68; *p* < 0.001). This observation may be related to the changes in the recommendations of the clinical guidelines, which from 2016 adjusted the titration targets of some drugs, such as beta-blockers. With regard to the renin–angiotensin system inhibitors (ACEi, ARB or ARNI), the initial doses were also higher in the group corresponding to the period 2011–2016 (0.59 vs. 0.49; *p* < 0.001), although after one year of follow-up both groups reached similar levels (0.73 vs. 0.72; *p* = 0.738), suggesting a tendency to equalize the degree of therapeutic optimization over time. The doses of mineralocorticoid receptor antagonists (MRA) remained stable in both periods, at both initial consultation and after one year, with no statistically significant differences (first consultation: 0.48 vs. 0.48; after one year: 0.49 vs. 0.50). Lastly, loop diuretics showed a higher dose in the patients corresponding to the period 2011–2016, at both initial consultation and after one year, probably driven by the individual congestive state of the patients.

### 3.4. Left Ventricular Ejection Fraction (LVEF) at One Year

**Figure 1:**
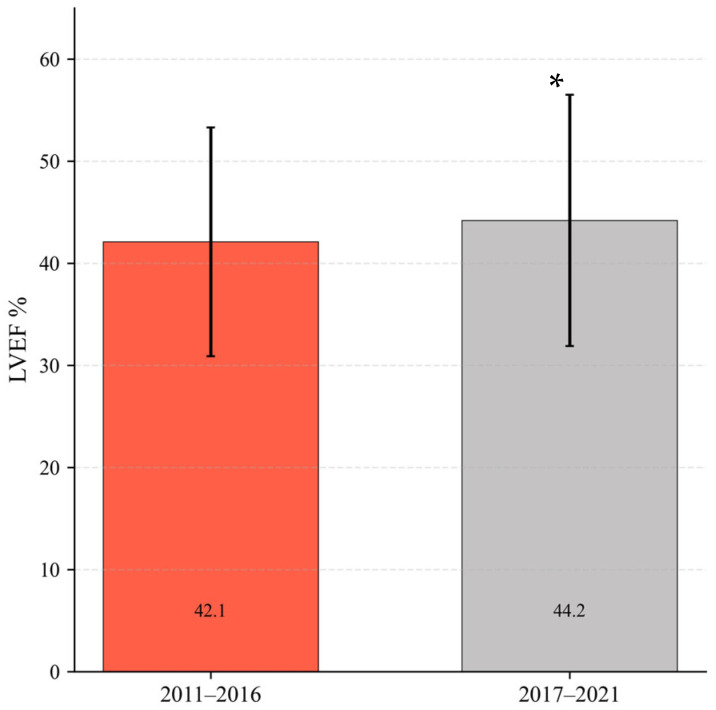
LVEF (%) after one year of follow-up. Data is presented as mean ± SD. * indicates *p* < 0.05.

The analysis was conducted on a total cohort of 1022 patients, with 618 individuals included during the period from 2011 to 2016 and 404 patients included during the more recent period from 2017 to 2021. Significant improvement of LVEF at one year was observed in the patients corresponding to the period 2017–2021, with mean values of 44.3 ± 11.2% versus 42.1 ± 12.3% in the patients corresponding to the period 2011–2016 (*p* = 0.005), suggesting an association between therapeutic optimization and improved ventricular function (Figure 1).

### 3.5. Implanted Devices

A cohort of 1217 patients who were not device carriers at the start of follow-up was analyzed. Of the total, 9.7% (*n* = 118) received an implantable cardioverter–defibrillator (ICD) and 1.2% (*n* = 15) received cardiac resynchronization therapy (CRT). The analysis includes patients receiving implants not only at the index center but also at any hospital worldwide.

**Figure 2:**
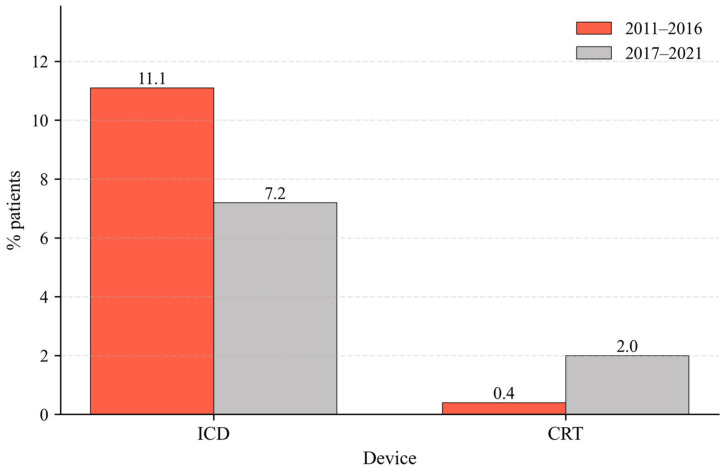
Indication of implantable devices in patients with no previous device (*n* = 1217). Data is presented as percentage.

The proportion of patients that received an implantable cardioverter–defibrillator was smaller in the group corresponding to 2017–2021 (7.2%) than in the group corresponding to 2011–2016 (11.1%) (*p* = 0.016) (Figure 2). In the univariate analysis, patients who received an ICD were significantly younger (63.1 vs. 66.2 years; *p* = 0.005), with a higher proportion of males (85.6% vs. 72.5%; *p* = 0.005) and a higher prevalence of ischemic heart disease (72.0% vs. 39.9%; *p* < 0.001) compared to those without a device. Multivariate analyses confirmed that the study period (2017–2021 vs. 2011–2016) was independently associated with a higher probability of ICD implantation (HR 0.46; 95% CI: 0.265–0.816; *p* = 0.008) (Table 4). This association persisted even after adjusting for age, sex, ischemic etiology, NYHA class, left ventricular ejection fraction (LVEF), and pharmacological treatment.

With regard to CRT, an increase was noted in the more recent group (2.0% vs. 0.4%; *p* = 0.005) (Figure 2). The profile of implanted patients was characterized by older age (72.2 vs. 65.8 years; *p* = 0.032). After adjusting for age, sex, etiology, functional class (NYHA), left ventricular ejection fraction (LVEF), and pharmacological treatment, the study period remained a significant predictor for receiving CRT (HR 5.61; 95% CI: 1.34–23.4; *p* = 0.018) (Table 4).

### 3.6. Survival at Two Years

**Figure 3:**
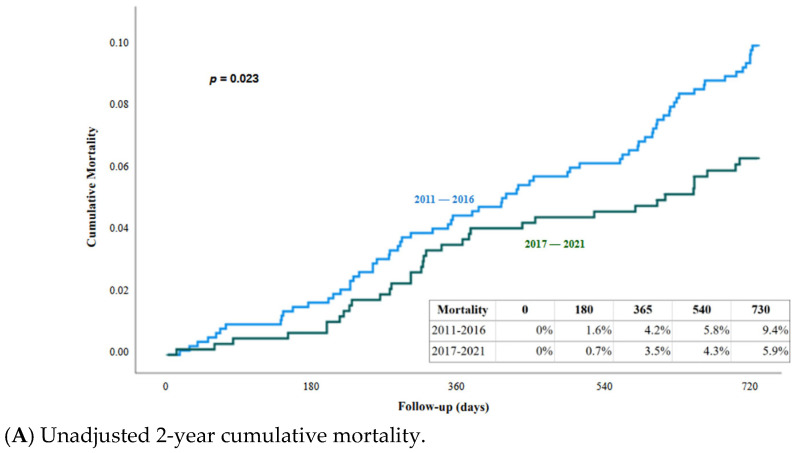

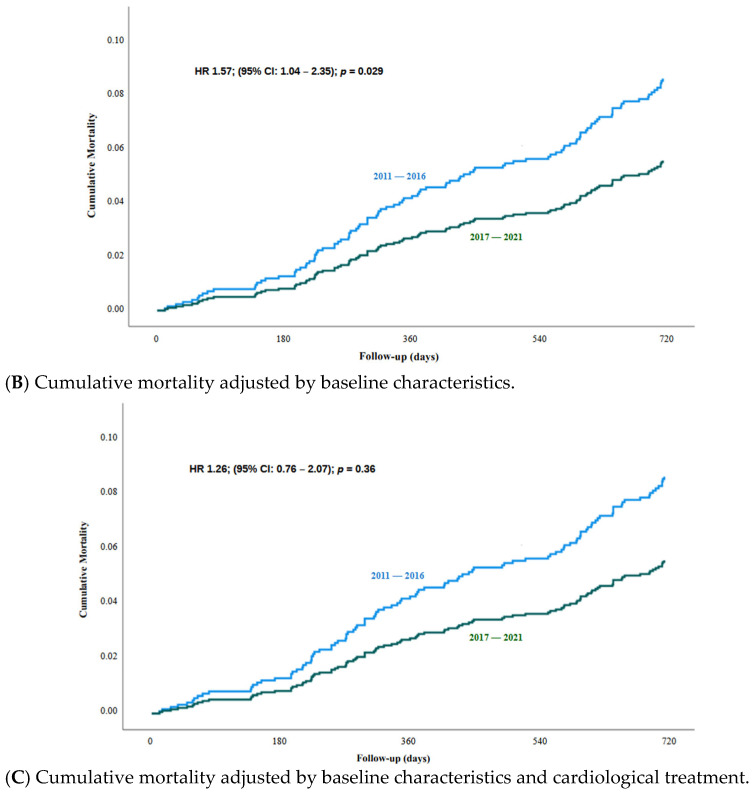
Actuarial mortality curve (Kaplan–Meier). (**A**) Unadjusted; (**B**) adjusted by baseline characteristics; (**C**) adjusted by baseline characteristics and cardiological treatment.

Complete follow-up was considered as 730 days ± 30 days or until death. A total of 61 patients did not meet this criteria, and are considered as lost to follow up (4.5%). The mean follow up for the complete population was 696 days ± 115. Survival analysis based on the Kaplan–Meier curves showed significant improvement of patient survival at two years in the group in which first consultation corresponded to the period 2017–2021 compared to 2011–2016. The cumulative mortality rate after the second year was 9.4% (95% CI: 7.3–11.5) in the group corresponding to 2011–2016, versus 5.9% (95% CI: 4.0–7.8) in the group corresponding to 2017–2021, the difference being statistically significant (log-rank test, *p* = 0.023) (Figure 3A).

To identify the determining factors of this improvement, two Cox regression models were applied. Firstly, a clinical model adjusting the baseline characteristics (sex, age, NYHA class, etiology, and LVEF) show that patients from the period 2011–2016 have a significant higher mortality risk than the 2017–2021 group (HR 1.57; 95% CI: 1.04–2.35; *p* = 0.029) (Figure 3B). In addition, age (HR 1.04; 95% CI: 1.02–1.06; *p* = 0.001) and NYHA class (HR 1.59; 95% CI: 1.12–2.26; *p* = 0.010) were significant clinical predictors of mortality (Table 5). When adjustment was made not only by baseline characteristics but also by baseline treatments (Model 2), the survival difference between the two periods was no longer statistically significant (HR 1.26; 95% CI: 0.76–2.07; *p* = 0.362). This suggests that the observed survival benefit in the 2017–2021 group was primarily driven by the introduction of ARNI, and maybe also by SGLT2i therapy, which remained an independent protective factor (Figure 3C) (Table 5).

## 4. Discussion

In this real-world, single-center observational study spanning more than a decade, we observed a significant improvement in two-year survival among patients with heart failure with reduced ejection fraction (HFrEF) managed in the most recent period (2017–2021) compared with those first evaluated between 2011 and 2016. This survival benefit, in which mortality decreased from 9.4% to 5.9% (*p* = 0.023), was evident in unadjusted analyses and persisted after adjustment for major baseline clinical characteristics. This indicates that temporal improvements in outcomes were not explained by differences in patient demographics or disease severity at presentation, as the baseline profile (characterized by an age close to 70, male predominance, and high prevalence of ischemic heart disease, arterial hypertension, and diabetes mellitus) remained stable and consistent with previous HFrEF registries [14], reinforcing the comparability of the analyzed groups.

When survival was adjusted for pharmacological treatment, the correlation between the time period and mortality was no longer statistically significant, suggesting that the observed improvement in survival is strongly associated with changes in medical therapy over time. This attenuation of the period effect supports the hypothesis that advances in heart failure management have played a relevant role in improving outcomes, as the most recent study period was characterized by a substantial increase in the use of guideline-directed disease-modifying therapies, specifically the progressive incorporation of sacubitril/valsartan (ARNI) and SGLT2 inhibitors following updates to international guidelines [6,7,8,9,15]. While initial doses of certain drug classes were higher in the earlier cohort, potentially due to higher baseline systolic blood pressure [16], the more recent group achieved effective titration during follow-up; furthermore, the lower utilization of loop diuretics in the 2017–2021 period likely reflects the enhanced natriuresis provided by ARNI and SGLT2i, which facilitates better congestion management [17].

These multivariate models were not designed to assess the independent prognostic effect of individual drug classes, but rather to explore whether temporal association with mortality persisted after accounting for changing treatment patterns. From this perspective, the loss of significance of the period variable after treatment adjustment supports the hypothesis that advances in heart failure management have played a relevant role in improving outcomes in routine clinical practice.

In parallel with pharmacological optimization, patients managed in the more recent period exhibited a significant improvement in left ventricular ejection fraction at one year (44.3% vs. 42.1%; *p* = 0.005). These findings are consistent with the reverse remodeling phenomena documented with contemporary therapies like ARNI, which can increase ejection fraction by 9% to 13% [18,19,20]. This improvement was associated with a subsequent reduction in device utilization: we observed a significant decrease in the prescription of implantable cardioverter–defibrillators (ICD), as many patients improved beyond the indication threshold. Conversely, the increase in cardiac resynchronization therapy (CRT) reflects both the expansion of clinical indications in recent guidelines [21] and the removal of local administrative barriers.

Overall, the significant improvement in survival observed over time in this cohort reinforces the clinical value of structured follow-up and active titration strategies in line with international guidelines [6]. These findings provide robust real-world evidence that advances in heart failure therapy have successfully translated into superior outcomes and enhanced ventricular recovery in routine practice, demonstrating that the synergy between broader access to contemporary drugs, meticulous dose adjustment, and improved patient adherence results in a tangible reduction in mortality and a significant improvement in LVEF.

## 5. Conclusions

This study demonstrates a significant improvement in two-year survival among patients with HFrEF over the last decade. This benefit was independent of baseline clinical characteristics but was attenuated after adjusting for pharmacological treatment, supporting the assumption that the observed prognostic improvement is primarily linked to the progressive adoption of contemporary disease-modifying therapies, particularly ARNI and SGLT2i. Although the observational nature of this real-world study precludes definitive causal inference, the results clearly suggest that the systematic application of therapies guided by evolving guidelines and active titration strategies has resulted in superior clinical outcomes and improved ventricular recovery. These findings highlight the critical role of pharmacological optimization and its association with an improved prognosis of heart failure at the population level in routine clinical practice.

### Limitations

The present study has a number of limitations inherent to its retrospective observational design. Firstly, the lack of randomization does not allow us to draw firm causal relationships between the prescribed treatments and the clinical observed outcomes. Therefore, these findings should be interpreted as descriptive of temporal trends in the treatment of heart failure in the real world, rather than as direct evidence of treatment efficacy.

Secondly, the use of electronic records may have introduced information bias due to incomplete registries of key variables. Furthermore, although multivariate analyses were adjusted for baseline characteristics, residual confounding by measuring factors, such as improvements in the organization of care, follow-up intensity and patient adherence, cannot be completely excluded. In addition, the study did not include data on quality of life, which is relevant clinical information.

Moreover, an important limitation is that pharmacological treatments were analyzed as baseline variables rather than time-dependent exposures, which might underestimate their true prognostic impact over the follow-up period. Regarding diagnostic tools, biomarkers such as NT-proBNP and advanced echocardiographic parameters were not consistently available during the earlier years of the study (2011–2016). This limitation in data restricts the accuracy of ventricular remodeling characterization and prevents a comprehensive comparison of prognostic markers across all cohorts.

Finally, the inclusion period ended in 2021, coinciding with publication of the latest European guidelines on heart failure. Therefore, our results may underestimate the current degree of implementation of the quadruple neurohormonal therapy as the new standard of care.

Although the attenuation of the ‘period effect’ after adjusting for these drugs suggests that they contributed to improved survival, this observation remains a hypothesis due to the possible influence of the unmeasured factors mentioned above.

## Figures and Tables

**Table 1:** Baseline characteristics.

	2011–2016 (*n* = 759)	2017–2021 (*n* = 604)	*p*	Standardized Difference
Age (years)	66.6 ± 10.9	66.1 ± 11.4	0.30	5.3
Male sex	574 (75.4)	456 (75.5)	0.95	0.23
SBP (mmHg)	132.3 ± 21.5	125.6 ± 20	**<0.001**	32.2
Creatinine (mg/dL)	1.1 ± 0.6	1.2 ± 0.8	0.09	10.2
GFR (ml/min/m^2^)	73.3 ± 24.2	71.3 ± 25.7	0.15	7.8
Active smoker	338 (44.6)	265 (45.5)	0.59	1.8
Arterial hypertension	532 (70.1)	404 (68.8)	0.60	2.8
Diabetes mellitus	288 (37.9)	236 (41)	0.25	6.3
Dyslipidemia	497 (65.6)	422 (72.8)	**0.005**	15.4
Ischemic heart disease	330 (43.5)	278 (46)	0.34	5.0
LVEF (%)	32.1 ± 8.2	32.6 ± 8.3	0.24	6.1
NYHA > II	65 (8.3)	33 (5.5)	**0.03**	10.9
BMI (kg/m^2^)	28.8 ± 4.8	29.1 ± 5.7	0.29	5.7
Underweight	5 (0.7)	3 (0.5)	0.81	-
Normal weight	151 (20.1)	119 (21.1)		
Overweight	332 (44.3)	236 (41.8)		
Obese	262 (34.9)	207 (36.6)		

Data are expressed as frequency (percentage) or mean ± standard deviation. SBP: systolic blood pressure; GFR: glomerular filtration rate; LVEF: left ventricular ejection fraction; BMI: body mass index.

**Table 2:** Pharmacological treatment of the patients in group 1 (2011–2016) and group 2 (2017–2021) at first consultation and after one year of follow-up.

	2011–2016 (*n* = 759)	2017–2021 (*n* = 604)	*p*
**First consultation**			
ACEi	595 (78.8)	280 (47)	**<0.001**
ARB	141 (18.6)	38 (11.2)	**0.002**
ARNI	1 (0.1)	255 (42.8)	**<0.001**
ACEi, ARB or ARNI	737 (97.1)	573 (94.8)	**0.011**
Beta-blockers	740 (97.5)	562 (94.3)	**0.003**
MRA	424 (55.9)	350 (58.7)	0.29
SGLT2i	0	145 (24.3)	**<0.001**
**Consultation 1 year**			
ACEi	565 (74.5)	223 (37.4)	**<0.001**
ARB	163 (21.5)	116 (19.5)	0.35
ARNI	8 (1.1)	238 (39.5)	**<0.001**
ACEi, ARB or ARNI	735 (96.8)	569 (94.4)	**0.025**
Beta-blockers	740 (97.6)	561 (93.8)	**<0.001**
MRA	434 (57.3)	386 (64.8)	**0.005**
SGLT2i	0	216 (36.6)	**<0.001**

Data presented as frequency (percentage). ACEi: angiotensin-converting enzyme inhibitors; ARB: angiotensin II receptor blocker; ARNI: angiotensin receptor–neprilysin inhibitor; MRA: mineralocorticoid receptor antagonists; SGLT2i: sodium–glucose cotransporter 2 inhibitors.

**Table 3:** Cardiological drug doses (with respect to recommended target dose).

Drug	Initial Dose		*p*	Dose at One Year		*p*
	2011–2016	2017–2021		2011–2016	2017–2021	
Beta-blockers	0.72 ± 0.40	0.61 ± 0.36	**<0.001**	0.89 ± 0.47	0.68 ± 0.38	**<0.001**
ACEi/ARB/ARNI	0.59 ± 0.39	0.49 ± 0.31	**<0.001**	0.73 ± 0.32	0.72 ± 0.32	0.73
MRA	0.48 ± 0.13	0.48 ± 0.14	0.87	0.49 ± 0.18	0.50 ± 0.18	0.37
Loop diuretics	1.19 ± 0.68	0.95 ± 0.64	**<0.001**	1.23 ± 0.7	0.98 ± 0.64	**<0.001**

Results expressed as mean ± SD. ACEi: angiotensin-converting enzyme inhibitors; ARB: angiotensin II receptor blocker; ARNI: angiotensin receptor–neprilysin inhibitor; MRA: mineralocorticoid receptor antagonists.

**Table 4:** Multivariate analysis of predictors of device implantation (logistic regression).

	Variable	Hazard Ratio	95% CI	*p*
**ICD**				
	Study period	0.465	0.265–0.816	**0.008**
	Sex	0.509	0.295–0.879	**0.015**
	Age	0.972	0.955–0.990	**0.002**
	Primary etiology	0.857	0.789–0.932	**0** **.000**
	NYHA	1.627	1.117–2.370	**0.011**
	Diagnostic LVEF	1.006	0.982–1.031	0.624
**CRT**				
	Study period	5.609	1.345–23.394	**0.018**
	Sex	0.556	0.144–2.145	0.394
	Age	1.063	0.999–1.132	0.054
	Primary etiology	1.083	0.943–1.244	0.257
	NYHA	1.300	0.475–3.560	0.610
	Diagnostic LVEF	0.995	0.933–1.062	0.891

LVEF: Left ventricular ejection fraction.

**Table 5:** Survival analysis (Cox regression) for 2-year mortality.

	Variable	Hazard Ratio	95% CI	*p*
**MODEL 1** **(Clinical)**				
	Study period	1.571	1.047–2.357	**0.029**
	Sex	0.420	0.242–0.730	**0.002**
	Age	1.037	1.016–1.059	**0.001**
	Primary etiology	0.994	0.931–1.061	0.857
	NYHA	1.587	1.115–2.260	**0.01** **0**
	Diagnostic LVEF	1.002	0.978–1.026	0.894
**MODEL 2** **(Clinical + Treatment)**				
	Study period	1.260	0.767–2.072	0.362
	Sex	0.399	0.229–0.695	**0.001**
	Age	1.034	1.012–1.056	**0.002**
	Primary etiology	0.987	0.924–1.054	0.693
	NYHA	1.506	1.042–2.177	**0.029**
	Diagnostic LVEF	1.003	0.979–1.028	0.792
	ARNI baseline	0.365	0.135–0.985	**0.047**
	SGLT2i baseline	0.475	0.175–1.292	0.145

LVEF: left ventricular ejection fraction; ARNI: angiotensin receptor–neprilysin inhibitor; SGLT2i: sodium–glucose cotransporter 2 inhibitors.

## Data Availability

The original contributions presented in this study are included in the article. Further inquiries can be directed to the corresponding authors.
